# Community-Based Interventions to Improve the Control of Non-Communicable Diseases in Underserved Rural Areas in Brazil: A Before-and-After Study

**DOI:** 10.3389/fphar.2021.644599

**Published:** 2021-04-14

**Authors:** Jéssica Caline Lemos Macedo, Vivian Carla Honorato dos Santos de Carvalho, Taciana Borges Andrade Cortes, Daniela Arruda Soares, Sóstenes Mistro, Clavdia Nicolaevna Kochergin, Davi Rumel, Marcio Galvão Oliveira

**Affiliations:** ^1^ Master’s Program in Collective Health, Multidisciplinary Health Institute, Federal University of Bahia, Vitória da Conquista, Brazil; ^2^ Multidisciplinary Health Institute, Federal University of Bahia, Salvador, Brazil; ^3^ Department of Community Health, School of Medicine of the Municipal University of São Caetano Do Sul, São Caetano Do Sul, Brazil

**Keywords:** primary health care, hypertension, diabetes, glycated hemoglobin, rural population

## Abstract

**Background:** Hypertension and diabetes mellitus are the second and third highest leading causes of disability-adjusted life-years (DALY), respectively, in Brazil. The clinical outcomes of chronic diseases are influenced by various factors. Therefore, there is a need for multifaceted interventions to achieve a decrease in the rate of DALY, with a better control of these diseases.

**Objective:** To verify whether sustainable long-term interventions, such as health worker training and provision of health education to the patients, contribute to health improvements in patients with hypertension and diabetes from rural communities.

**Methods:** Over a 6 month period, educational and medical interventions were provided to optimize the treatment of hypertension and diabetes. Furthermore, blood pressure and glycated hemoglobin (HbA1c) measurements were taken at baseline and after the interventions.

**Results:** The monitored hypertensive patients (*n* = 276) had a reduction of 13.4 mmHg (*p* = 0.021) and 5.8 mmHg (*p* < 0.001) in mean systolic and diastolic blood pressure, respectively. Diabetic patients who were followed-up (*n* = 71) achieved a 0.55% (*p* = 0.185) reduction in HbA1c level. The desired blood pressure level (<140/90 mmHg) was achieved in 38.8% of patients with hypertension, whereas the desired level of HbA1c (<7.0% for adults and <8.0% for the elderly) was achieved in 16.9% of patients with diabetes; in addition, 38.0% had a reduction of HbA1c of at least 1%.

**Conclusion:** The results showed that the interventions improved the blood pressure and HbA1c levels in patients with hypertension and diabetes from rural communities in a municipality in Northeast Brazil.

## Introduction

The prevalence of non-communicable diseases (NCDs) has been increasing worldwide and this significantly impacts the health systems (Flor et al., 2020). In Brazil, hypertension and diabetes are the second and third leading causes of disability-adjusted life-years (DALY) (Costa et al., 2017; Nascimento et al., 2020), a measure that combines information about premature death and disability due to a condition (Nascimento et al., 2020). The total number of deaths and DALY are increasing due to the aging of the population and the increased risk exposure; however, the DALY rates have decreased in Brazil, possibly reflecting an improvement in access and quality of health care (Nascimento et al., 2020).

The investment in the implementation and improvement of strategies to provide patients with NCDs in the primary health care settings has yielded good results by reducing the need for specialized care and health system expenses (Flor et al., 2020; American Diabetes Association, 2020). Given that a variety of factors can influence the clinical outcomes of chronic diseases, multifaceted interventions are required to achieve the desired results, either at the individual level with a timely medical diagnosis and appropriate drug treatment, or at the community level, with health education and monitoring (He et al., 2017; Flor et al., 2020; Silva-Tinoco et al., 2020). In other words, multicomponent community intervention programs are more successful than interventions based on a single activity (Philip et al., 2018).

The social determinants of health, including economic, environmental, political, and social conditions, also significantly affect the control of NCDs (American Diabetes Association, 2020). This is especially important for patients from rural communities because in addition to the factors mentioned above, this population encounters cost barriers when seeking transportation to access health services (Flor et al., 2020).

The demographic profile of rural environments has changed over time, with a higher proportion of older patients than in the urban areas. As birth rate decreases, the rural exodus of young people in search of better living conditions to urban areas increases, triggering the aging of rural populations, and consequently, an increase in the need for chronic diseases care (Crhisten, 2016).

The Brazilian Unified Health System (*Sistema Único de Saúde*-SUS) still faces organizational problems that are limiting access to adequate treatment of patients (Mendes et al., 2012). This is especially important for rural populations that have budgetary restrictions, which affect access to private health plans, and enhance dependency on public policies (Pessoa et al., 2018).

Therefore, we conducted this study considering 1) the scarcity of intervention studies of patients with hypertension and diabetes from rural communities in Brazil and 2) the greater need for access to health care in this population.

Thus, the aim of the study was to evaluate whether multicomponent interventions would improve the control of blood pressure (BP) and glycated hemoglobin (HbA1c) level in four rural areas in Brazil where patients need more health care.

## Methods

### Procedures

A before-and-after uncontrolled study was conducted in which the baseline and post-intervention BP (for hypertensive patients) and HbA1c (for diabetes patients) levels were compared, and the baseline levels for each patient served as the control for the subsequent measures. The study was conducted in four basic health units of different rural communities in a municipality in northeastern Brazil, from September 2019 to March 2020.

This study implemented a 6 months multicomponent intervention program. This included home visits by community health workers (CHWs), health worker training, and point-of-care monitoring of the BP and HbA1c levels during patients’ meetings (including during health fairs and in patients’ attendance groups). Furthermore, health education for patients and their families to manage chronic NCDs were also provided.

The group-based self-management education was prioritized because it was more cost-effective (Steinsbekk et al., 2012), and at least two groups of patients were followed-up per month in each unit. Because it was a rural area, the patients’ homes were very distant from one another; thus, each shift (4 h) of work by the research team contemplated on average, only three home visits. The health team activities in the waiting room of the health units were limited to the days of care of patients with hypertension and/or diabetes.

Due to the coronavirus disease (COVID-19) pandemic, it was no longer possible to bring patients together in collective activities because they belonged to the risk group of COVID-19. Thus, the study ended 45 days before the forecast, which generated a loss of follow-up.

The research team that implemented the interventions in the four health units comprised of 20 people (8 researchers and 12 undergraduate students), who were trained prior to the beginning of the data collection. Each team member received a tablet containing the Kobo Tool Box® software with socioeconomic, clinical questions and BP and HbA1c measurements.

Medical professionals, nurses, and CHWs also underwent training to better manage the patients with hypertension and diabetes during the follow-up. The training took place separately for each category of health workers within a 6 h workload.

The inclusion criteria were all the patients who were able to be reached during home visits, health fairs, and patients’ attendance groups in the 6 month period. Patients should be over 18 years old and who were registered in health units and were diagnosed with hypertension (BP ≥ 140/90 mmHg) (Williams et al., 2018) and/or uncontrolled diabetes mellitus (HbA1c above the therapeutic targets: >7.0% for adults and >8.0% for patients aged ≥65 years) (American Diabetes Association, 2020). During the study period, the research team assisted the CHWs and health teams to identify patients with uncontrolled conditions through home visits, health fairs, and patients’ attendance groups and served as mediators between the patients and physicians, to arrange follow-up appointments.

For hypertensive patients, three BP measurement were performed–one on each arm–with a repeat measurement on the arm with a higher value using the Omron HEM-7113 automatic BP measuring device. These measurements were obtained while the patients were seated after resting for at least 5 min (Malachias et al., 2016). The mean of the two highest values were calculated.

For diabetic patients, HbA1c levels were measured using the portable Abbott Afinion™ two Analyzer, a point-of-care device that allows for instant evaluation without needing to visit a clinical laboratory. In a study, “the Cost-Effectiveness study of Point-of-Care A1C Tests in a Primary Care Setting,” because the point-of-care testing of HbA1c levels resulted in an increase in the rate of diabetes control, the higher purchase costs of the point-of-care testing compared to that of laboratory tests, were reportedly offset by the savings from the reduction in diabetes-related complications and hospitalizations (Rosa et al., 2021).

BP and HbA1c levels were measured again within 3 months of the intervention, for each patient. The outcomes were controlled hypertension (<140/90 mmHg) and diabetes (HbA1c <7% for adults; <8% for older persons).

Patients received guidelines to promote self-care, attain a healthy lifestyle, adhere to treatment, and on the need for more frequent follow-ups for new clinical measures. Furthermore, patients with altered HbA1c levels and BP were referred for consultation with a family physician, during which adjustments were made to the drug treatment (whenever necessary) along with reinforcement of information on living a healthy lifestyle, treatment adherence, and self-care, based on the study protocol.

### Study Site Context

Brazil has a public health model—the SUS—that guarantees health as a right of citizenship and is an organized healthcare network; specifically, it is configured in the articulated actions of services with different functions to meet the healthcare needs of the population. The main gateway to this healthcare network should be the primary care setting, where most health issues that people face should be addressed. Each basic health unit has a coverage area where residents are registered (Giovanella and Mendonça, 2012). There are still many organizational failures of the SUS that generate dissatisfaction within the population due to the difficulty of carrying out tests, long waiting times before access to specialists, and availability of medicines (Mendes et al., 2012).

The municipality under study has 17 Family Health Units in the rural area, of which 4 units were chosen by convenience. These units, according to the Municipal Health Department data, are reference centers for approximately 18,376 people and have 2,788 hypertensive patients and 565 with diabetes. Each health unit is composed of one each of a physician, nurse, nursing assistant and/or technician, dentist, and oral health assistant or technician, and 6 to 11 CHWs.

### Statistical Analysis

Data analysis was performed using Stata version 15.0 (Stata Corporation, College Station, United States). Sociodemographic and disease variables were described as absolute and relative frequency. Pearson’s Chi-square test was applied to assess the association between sociodemographic variables and the presence of controlled diseases (hypertension and diabetes), with a significance level set at *p* < 0.05. Differences between the mean BP and HbA1c before and after the interventions were assessed using the Kruskal-Wallis non-parametric test; because the Shapiro-Wilk test demonstrated that there was no normality in the numerical variables.

### Ethical Aspects

The study was approved by the Ethics Committee on Research in Human Beings of the Multidisciplinary Institute in Health–Campus Anísio Teixeira (Opinion number: 3.357.963).

Informed consent was waived from the patients because the research could not practicably be conducted without a waiver and involved no more than minimal risk.

## Results

The baseline study comprised 638 patients who were diagnosed with uncontrolled hypertension (*n* = 556) and/or diabetes (*n* = 191) (Table 1).

**TABLE 1:** Sociodemographic characteristics and presence of disease of the study population.

Variables	N	%
Sex		
Male	208	32.6
Female	430	67.4
Age (years)		
<40	42	6.6
40 to 59	246	38.5
60 to 79	301	47.2
≥80	49	7.7
Skin color		
White	125	19.6
Black	163	25.5
Brown	317	49.7
Other	33	5.2
Literacy		
Illiterate	227	36.3
Literate	399	63.7
Marital status		
Single/divorced/widowed	226	35.4
Married/lives with partner	412	64.6
Working status		
None	181	28.4
Work	98	15.3
Retired	359	56.3
Monthly per capita income in dollar^a^		
<$ 53.19	180	29.3
≥$ 53.19	435	70.7
Diagnosis of hypertension and diabetes		
Hypertension only	372	58.3
Diabetes only	32	5.0
Hypertension and diabetes	234	36.7

^a^ US$ 1 = R$ 5.64 on October 27, 2020.

NCDs, non-communicable diseases.

Follow-up was completed for 276 (49.6%) hypertensive patients and 71 (37.2%) diabetic patients. The study was interrupted due to the COVID-19 pandemic that led to a greater loss of intense follow-up of diabetes patients, as HbA1c had to be measured every 3 months.

Following intervention, the systolic BP (SBP) of patients had a mean reduction of 13.4 mmHg (*p* = 0.021) and the diastolic BP (DBP) had a mean reduction of 5.8 mmHg (*p* < 0.001). Regarding HbA1c levels of diabetic patients, the mean reduction was 0.55% (*p* = 0.185) (Table 2).

**TABLE 2:** Results before and after the intervention for the 276 hypertensive and 71 diabetic patients.

Mean	Baseline	95% confidence interval	After intervention	95% confidence interval	Reduction	*p*-value
SBP (mmHg)	162.2	160.1–164.4	148.8	146.2–151.6	13.4	0.021
DBP (mmHg)	90.0	88.4–91.5	84.2	82.6–85.8	5.8	<0.001
HbA1c (%)	10.02	9.7–10.4	9.47	9.1–9.80	0.55	0.185

DBP, diastolic blood pressure; SBP, systolic blood pressure.

BP was controlled (<140/90 mmHg) in 107 (38.8%) patients; while the SBP decreased by ≥ 10 mmHg in 156 (56.5%) patients and DBP decreased by ≥ 5 mmHg in 141 patients (51.1%). HbA1c levels were controlled (<7.0% for adults or <8.0% for older persons) in 12 (16.9%) patients, and there was a reduction of ≥1% in HbA1c levels in 27 (38.0%) patients.

As shown in Table 3, a per capita income <$ 53.19 was associated with greater hypertension control (*p* = 0.017) and being male was associated with a higher diabetes control (*p* = 0.001).

**TABLE 3:** Assessment of hypertension and diabetes control by sociodemographic characteristics.

Variables	Hypertension					Diabetes				
	Not controlled		Controlled		*p* value	Not controlled		Controlled		*p* value
	N	%	N	%		N	%	N	%	
Sex					0.737					0.001
Male	57	62.6	34	37.4		8	53.3	7	46.7	
Female	112	60.5	73	39.5		51	91.1	5	8.9	
Age (years)					0.387					0.577
<40	4	36.4	7	63.6		6	100.0	0	0.0	
40 to 59	64	62.7	38	37.3		24	85.7	4	14.3	
60 to 79	82	61.6	51	38.4		26	78.8	7	21.2	
≥80	19	63.3	11	36.7		3	75.0	1	25.0	
Skin color					0.267					0.142
White	33	66.0	17	34.0		10	83.3	2	16.7	
Black	55	67.9	26	32.1		17	85.0	3	15.0	
Brown	69	55.6	55	44.4		30	85.7	5	14.3	
Other	11	55.0	9	45.0		1	33.3	2	66.7	
Literacy					0.441					0.287
Illiterate	64	59.3	44	40.7		16	76.2	5	23.8	
Literate	101	63.9	57	36.1		39	86.7	6	13.3	
Marital status					0.710					0.524
Single/divorced widowed	56	59.6	38	40.4		20	87.0	3	13.0	
Married/lives with partner	112	61.9	69	38.1		38	80.8	9	19.2	
Working status					0.196					0.737
None	42	53.2	37	46.8		21	87.5	3	12.5	
Work	24	68.6	11	31.4		7	77.8	2	22.2	
Retired	102	63.3	59	36.7		30	81.1	7	18.9	
Monthly per capita income in dollar*					0.017					0.062
<$ 53.19	38	50.0	38	50.0		20	95.2	1	4.8	
≥$ 53.19	125	65.8	65	34.2		36	76.6	11	23.4	
Diagnosis of hypertension and diabetes					0.815					0.374
Hypertension only	106	60.6	69	39.4		—	—	—	—	
Diabetes only	—	—	—	—		11	91.7	1	9.3	
Hypertension and diabetes	62	62.0	38	38.0		47	81.0	11	19.0	

## Discussion

Our study showed a significant reduction in the mean BP, but not in HbA1c level, in patients who completed the follow-up. Several studies indicate that considerable decreases in morbidity and premature mortality correlate with reductions in BP among hypertensive patients (Williams et al., 2018) and with glycemic control in diabetes patients (Schnell et al., 2017).

However, the achievement of BP and HbA1c control remains low worldwide (Schnell et al., 2017; Williams et al., 2018). Our BP control results were superior to those of another intervention study conducted in a specialized outpatient clinic in Brazil, where BP control was achieved in 30% of the patients (Coelho et al., 2005). Another study conducted by our team in urban areas of two Brazilian cities found that hypertensive patients had better BP control at the end of the intervention than at baseline. Therefore, at 1 year of follow-up in the two cities, mean reductions of 4.2 mmHg (*p* < 0.001) and 1.9 mmHg (*p* < 0.01) in SBP and DBP occurred, respectively (*p* < 0.05) (Flor et al., 2020).

A decrease of 10 mmHg in SBP or 5 mmHg in DBP was associated with significant reductions in all major cardiovascular events (approximately 20%) and all-cause mortality from 10 to 15% (Williams et al., 2018). In our study, 56.5 and 51.1% of the patients achieved these reductions in SBP and DBP, respectively.

Considering the socioeconomic characteristics of the population in our study, an association was found between having lower income and hypertension control in patients (*p* = 0.017). This result suggests that the interventions had a higher impact on this population group because it led to a reduction in health inequalities more often faced by this group of people.

Regarding diabetic patients, being male was associated with a greater control of diabetes (*p* = 0.001). This result suggests that the active search of patients by the work team, the timely interventions, and the offer of point-of-care measurement of HbA1c were more important for this group of patients, since men seek less health services than women (Lima et al., 2011).

In our study the mean reduction in HbA1c levels following the intervention was not significant. This result may be related to the early interruption of follow-up of these patients due to the COVID-19 pandemic in Brazil. Nevertheless, some patients achieved the control of HbA1c levels, and a larger number achieved a reduction of at least 1% in HbA1c levels. This reduction is important because it is associated with significant decreases in microvascular complications and mortality (Krishnamurti and Steffes, 2001).

The American Diabetes Association (ADA) recommends that HbA1c tests be performed twice a year for patients who are within the metabolic control goal and every 3 months for patients with change in treatment and/or out-of-goal test results (American Diabetes Association, 2020). However, most people with diabetes do not undergo HbA1c monitoring as recommended by the ADA (Egbunike and Gerard, 2013). In addition to the lack of testing following HbA1c test requests, there is an additional challenge whereby the test results are withheld until the patient clinically consults with the health professionals, which leads to delays in the administration of therapeutic strategies (Egbunike and Gerard, 2013).

In the study conducted by our team in two Brazilian cities, HbA1c levels were reduced by 0.6% (*p* < 0.001) and 0.9% (*p* < 0.001), respectively. Furthermore, approximately 10.3 and 25.0% more patients achieved diabetes control than those at baseline in each of the cities (Flor et al., 2020). A study in South Africa showed that after 2 years of providing HbA1c point-of-care testing at a primary care clinic site, a 1.3% reduction in the mean HbA1c (*p* < 0.01) level was achieved (Motta et al., 2017). In our population, following intervention, approximately 17% of the diabetic patients achieved disease control compared to that at the baseline, and 38% achieved a reduction of at least 1% in HbA1c.

In our study, the positive results may also be related to the benefits of using a point-of-care device to measure HbA1c levels. This allowed examinations to be performed at the time of care with the immediate result of the measurement availed; hence, essential interventions for effective control, such as treatment changes and emphasis on lifestyle changes, could be performed instantly (American Diabetes Association, 2020). Furthermore, a study showed that although the unit cost of the HbA1c measurement per point-of-care device was higher than that measured in a laboratory, its use was associated with cost reduction in medical care (Schnell et al., 2017).

However, with this being a quasi-experimental study, it is important to note that this type of design has some intrinsic limitations, including 1) the absence of random samples and 2) the absence of a comparator group in relation to the group of differentiated participants with interventions. Another limitation of this study was the short time of intervention and loss of follow-up; therefore, studies with longer follow-up periods may show more robust results.

Nevertheless, even though the randomized trial is the gold standard in the evaluation of community intervention trials, practical and ethical issues argue in favor of the almost experimental study model. This is because the interventions needed to be applied to as many patients as possible by the work team, in a community in need.

The innovative process of this study facilitated access to multifaceted interventions by patients from rural communities that effectively improved their BP and HbA1c levels. Despite the short duration of the study, the interventions can be sustainable on the long term, as they were designed to support family health teams in identifying patients with uncontrolled hypertension and diabetes, in addition to promoting lifestyle changes, adherence to treatment, self-care, and optimization of therapy. Therefore, we strongly encourage that other multicomponent intervention studies with longer durations be conducted in patients with diseases such as NCDs, to aid health professionals in improving the care of these populations.

## Data Availability

The raw data supporting the conclusion of this article will be made available by the authors, without undue reservation.
